# Using health impact assessment (HIA) to understand the wider health and well-being implications of policy decisions: the COVID-19 ‘staying at home and social distancing policy’ in Wales

**DOI:** 10.1186/s12889-021-11480-7

**Published:** 2021-07-27

**Authors:** Liz Green, Kathryn Ashton, Sumina Azam, Mariana Dyakova, Timo Clemens, Mark A. Bellis

**Affiliations:** 1 Public Health Wales, Cardiff/Wrexham, Wales UK; 2 Department of International Health, Care and Public Health Research Institute – CAPHRI, Maastricht University, Maastricht, The Netherlands; 3 Department of Public Health and Life Sciences, Bangor University, College Road, Bangor, Wales UK

**Keywords:** Health impact assessment, COVID-19, Lockdown, Wales, Health and well-being, Public health

## Abstract

**Background:**

Health Impact Assessment (HIA) is promoted as a decision-informing tool by public health and governmental agencies. HIA is beneficial when carried out as part of policy development but is also valuable as a methodology when a policy is being implemented to identify and understand the wider health and well-being impacts of policy decisions, particularly when a decision needs to be taken rapidly to protect the population. This paper focusses on a HIA of the ‘Staying at Home and Social Distancing Policy’ or ‘lockdown’ in response to the COVID-19 pandemic in Wales conducted by the Welsh national public health institute. It describes the process and findings, captures the learning and discusses how the process has been used to better understand the wider health and well-being impacts of policy decisions beyond direct health harm. It also examines the role of public health institutes in promoting and using HIA.

**Methods:**

A HIA was conducted following a standard HIA five step process. A literature review was undertaken alongside 15 qualitative semi-structured interviews with key stakeholders, and relevant health and demographic data were collated. The results were triangulated and analysed to form a holistic assessment of the policy decision and its impacts.

**Results:**

A wide range of major health and well-being impacts of the lockdown in Wales were identified across the determinants of health, which included positive and negative social, economic, environmental and mental well-being impacts beyond the impact on direct health. Populations affected included children and young people, those on low incomes and women as well as those whose health has been directly impacted by COVID-19 such as older people. The work highlighted the benefit that HIA can bring in emphasizing impacts which can inform policy and shared learning with others.

**Conclusion:**

HIA is a largely underused tool to understand the impact of policy and political decisions, particularly when a decision has been taken at speed. This case study highlights how HIA provide evidence and information for advocacy and further work by public health institutes, health agencies and policy makers.

**Supplementary Information:**

The online version contains supplementary material available at 10.1186/s12889-021-11480-7.

## Background

Policy decisions made internally or externally to the health sector can have a significant impact on individual and population health and well-being [1–3]. These impacts can occur directly or indirectly via the pathways of the social determinants of health (SDOH) [4] which relate to wider social, economic and environmental factors that have an impact outside of the health system [5–7]. This includes for example, transport policies that can enable or inhibit active travel and physical activity, but can also have an impact on air quality and respiratory conditions [8–10]. Globally, national policy makers and politicians have a responsibility to protect the health and well-being of populations [11–13]. However, the potential wider health and well-being impacts are not specifically and routinely taken into consideration when making policy decisions [14–17], except for a few examples in which health and well-being is required in legislation to be considered as part of wider policy making [18–20].

Health Impact Assessment (HIA) is promoted as a decision-informing tool [21–23] which can support the development and implementation of policy decisions by identifying a broad and holistic health impact across the determinants of physical, social, environmental and mental health and well-being [16, 24]. HIA is a method which can be applied to systematically and flexibly to appraise a policy, plan or project or intervention [25, 26], acknowledged as a cornerstone of Healthy Public Policy-making [27] and includes the concept of ‘Health in All Policies’ (HiAP) [28]. This is an approach that seeks synergies between sectors, avoids harms to health and promotes equity [21, 28]. It is not the only form of impact assessment (IA) which can be carried out on policies, for example Social Impact Assessment [29], Mental Well-being Impact Assessment [30, 31] and Strategic Environmental Assessment [32] can all be utilised either legally or voluntarily. These all allow input from a wide range of stakeholders including the health sector, but the primary focus is on a particular area for example, mental health and well-being [31] or a or specific group with protected characteristics such as sex or religion. Health can also be included and considered to varying degrees within these and it has been noted that IAs can learn from each other to better integrate health and other considerations [33, 34]. HIA, however, provides a specific vehicle for the explicit consideration of health and wider well-being in a cross sectoral, participative and systematic way [21, 35–37].

HIAs can be carried out prospectively (before a decision has been taken), concurrently (as one is being implemented) or retrospectively (after it has been implemented and concluded) [21, 28]. It is advocated as a tool which is utilised most effectively prospectively in order to inform decision makers about the potential future anticipated positive or negative impacts that a policy decision can have, and identifies who may be affected by that decision [38–40]. The prospective approach is widely supported in the literature [41–46] with little being published about concurrent or retrospective HIAs which are less frequently carried out [47]. However, a recently published best practice principles suggest that these descriptions along with terminology such as rapid, intermediate and comprehensive should be dispensed with as they can cause confusion and be misleading amongst decision makers [35].

Whilst some papers discuss the effect of HIA in general on policy decision-making processes [38, 39], there is sparse literature internationally which discusses how HIAs can be utilised or practically applied to better understand the wide-ranging consequences of a policy decision once it has been agreed and is being, or has been, implemented [47]. This is an interesting missing component of HIA practice particularly where examples of unique or emergency events such as the COVID-19 pandemic would benefit from a HIA being carried out as it unfolds (or after the event has occurred) in order to learn from, and inform, future decision making processes. Some authors discuss the effectiveness of HIA in different sectors or have evaluated HIA effectiveness which are based on case studies [38, 39] and these detail the enablers such as the flexible process, stakeholder engagement and the learning which can be captured when carrying out a HIA whilst time and resources could be a challenge.

It has also been noted in other IA literature for example, in relation to disaster management planning that there is little research on this topic and that using IA in order to prepare for, and recover from, disaster or emergency events would be advantageous [48]. Furthermore, others have highlighted that the failure of some cities across the world to prepare for an unexpected event such as COVID-19 through an equity lens resulted in negative health impacts for many population groups [49]. Therefore carrying out an health and equity focused impact assessment such as HIA can be beneficial as it can ensure that evidence can be gathered, synthesized and published to systematically support future policy and decision-making, raise awareness of potential health and well-being implications (particularly when outside of the health care sector) and ensure mitigation is in place to address inequalities that may arise [15, 50]. Health agencies such as national and regional Public Health Institutes (PHIs), have a critical role in supporting this activity. For the purpose of this paper, a PHI is defined as ‘a government agency, or closely networked group of agencies, that provides science-based leadership, expertise, and coordination for a country’s or region’s public health activities.’ [51]. These agencies deliver essential public health functions including gathering health intelligence, support health protection and promote health and well-being [52–54]. These elements are also key components of HIA practice which identifies positive impacts for health promotion, and negative impacts which need to be mitigated for to protect health [25, 55, 56]. As such PHIs can be an important advocate and natural host for HIA activity with a focus on evidence based health equity [37]. To date, little has been researched and published to illustrate the role of PHIs in using HIA to promote and enable a preventive, Health in All Policies approach to inform decision-making, beyond their role in capacity building or advocacy for HIA [40, 52].

Wales is one of four United Kingdom nations and Welsh Government is devolved and therefore can make its own laws across a breadth of areas for example, health and social care and spatial planning [57]. Wales provides an unusual context for HIA with a dedicated Health Impact Assessment Support Unit (WHIASU) based in the World Health Organization (WHO) Collaborating Centre on Investment for Health and Well-being Directorate at Public Health Wales, the national public health institute, legislation to maximise well-being [18] and a public health law which requires HIAs to be carried out by public bodies such as Welsh Government, Public Health Wales, local Health Boards and local authorities in specific circumstances. This includes a consideration of major policies or plans such as planning policies [19]. WHIASU provides HIA advice, guidance and training and occasionally carries out HIAs which identify any health impact of important policies or events and shares learning with others. This has been highlighted as a positive factor in HIA capacity building [58, 59].

In April 2020, Public Health Wales carried out a HIA of the ‘The Health Protection (Coronavirus) (Wales) Regulations 2020’ [60] - commonly referred to as the ‘Staying at Home and Social Distancing’ policy (SAH Policy) in Wales due to the associated published guidance for the Welsh restrictions [61]. HIA methodology was chosen by the PHI for several reasons. HIA has a specific evidence based focus on identifying the cross sectoral health and well-being impacts and population groups who would be affected by the novel national policy, which had been drafted and implemented as speed. At that time, there was little or no evidence of how a worldwide modern pandemic would impact wider health and well-being and it has been highlighted that whilst there is now a wide range of evidence of the health and wider effects in Wales and across the world [62–66]. It would provide statistical and qualitative evidence to inform future action and this would be enhanced by internal and external engagement with key stakeholders such as the Welsh Local Government Association, the Children’s Commissioner for Wales, Healthy Working Wales teams. Others factors included the expertise of WHIASU [59] and the positive experience and influence of carrying out a previous HIA on the United Kingdom withdrawal from the European Union (‘Brexit’) [37]. Additionally, it would demonstrate leadership for HIA and HiAP by voluntarily carrying out HIA in advance of the enactment of Welsh Government legislation for HIA [19]. The work complemented and supported the organisational acute health protection response to control the transmission of the virus [67, 68] and existing work with government and local health boards. Furthermore, it enabled PHW, Welsh Government and other key stakeholders to ensure that any harms caused by ‘lockdown’ could be mitigated for, or considered along with positive impacts and opportunities, in future decisions during and post the pandemic. The HIA provided Wales with specific evidence to support targeted policy making and interventions during the pandemic recovery and renewal phases. The majority of the authors are employed by, or work with the national public health institute and carried out the HIA discussed in this paper.

The purpose of this paper is to add to the evidence base which policy and decision-makers and the HIA community can draw upon and utilise in the future to inform, promote or carry out similar HIAs. It aims to demonstrate how HIA can be implemented in practice to examine and better understand the impact of a rapid policy decision on health and well-being once it has been taken and has been, or is being, implemented. Using the example of ‘the ‘Staying at Home and Social Distancing’ policy (SAH Policy) in Wales which were implemented in response to the COVID-19 pandemic, this paper highlights how HIA has enabled health sector bodies for example, health boards and wider ‘non-health’ sector stakeholders, for example, local government with wide ranging responsibilities including spatial planning, economic development, transport and environment to understand the wider impact of the pandemic. It articulates how the policy has impacted the social determinants of health, well-being and inequalities in Wales and captures transferable learning from it to inform and support public health practitioners, decision and policy-makers nationally and globally. In addition, it raises awareness about the role of PHIs with respect to HIA and how they can use the HIA process to understand and explicitly communicate the wider societal harms and benefits, both inside and outside of the health care sector, to advocate for healthier populations and fairer societies.

## Methods

In 2020, Public Health Wales, undertook a HIA of the SAH Policy in Wales in response to the COVID-19 pandemic otherwise referred to as ‘lockdown’. It was carried out in real time whilst lockdown measures were in place. To date it is the only HIA of such a COVID-19 related measure with only one other screening example [69]. The lockdown measures implemented in Wales on the 24th March 2020 included a requirement to stay at home at all times except for a few exceptions, to work from home if you can and to ensure that social distancing from people not in your household was at least two metres at all times (Table 1) [60].

**Table 1:** An overview of The Health Protection (Coronavirus) (Wales) Regulations 2020′ implemented in Wales on 24th March 2020 [60]

The regulations:	
• provided Welsh Ministers, registered public health officials and police constables the right to detain people contaminated or infected with coronavirus.	
• required some business premises to close (those classed as non-essential such as leisure and hospitality) and required those allowed to remain open (those classed as essential, such as food retailers and supermarkets), to put specific measures in place to ensure adequate social distancing.	
• restricted individuals movements so that they were prohibited to leave the place they were living without a ‘reasonable excuse’. The regulations included examples of a ‘reasonable excuse’ for example, shopping for food, taking physical exercise once a day, obtaining medical assistance and travelling to a place of work where it was ‘not reasonable and practicable to work from home’.	
• closed places of worship, apart from in limited circumstances such as in relation to funerals.	
• required Natural Resources Wales (the environment agency for Wales), local authorities, National Park Authorities and the National Trust to close public footpaths and access land, where the use of a path or land posed a high risk of spreading coronavirus.	
• changed elements of planning restrictions. The UK Government also made regulations and changes in non-devolved areas, for example, for statutory sick pay, Universal Credit and other welfare benefit claims.	
Welsh Parliament also approved other health related legislation including some changes for example, to the regulations for Mental Health Tribunals, amended rules for social care standards. Although there was coordination in health policy across the UK in respect to addressing the pandemic (and the Chief Medical Officers worked closely to develop a shared evidence base for the four national governments), Welsh policy diverged in places from that of England. Welsh Government policy included secondary legislation, for example, closing all caravan parks in Wales to reduce people travelling to these in order to isolate or ‘lockdown’.	

The HIA assessed the impact of this policy across a wide range of determinants of health and identified those population groups who may have been disproportionately affected by lockdown in Wales. It did not assess the direct physical health impact of COVID-19 in relation to transmission rates, morbidity and mortality, but viewed the pandemic through a broader social determinants and inequalities lens [4]. It aimed to capture the wider potential or actual harms or benefits and unintended consequences which such lockdowns can have.

This HIA followed a standard five-step evidence based process [21, 25], which is depicted in Table 2. An additional supplementary table show this in more detail (see Additional Table 1).

**Table 2:** The HIA Process

HIA Step	Actions	
**1. Screening**	The wide ranging populations and determinants affected were identified and a Steering Group was established.	
**2. Scoping**	The scope of the HIA was defined with a clear focus on Wales. Methods decided upon were a literature review, collation of health intelligence data and interviews with key stakeholders.	
**3a. Appraisal –Evidence Gathering**	Literature Review	Carry out literature review and synthesise into summary to identify relevant qualitative and quantitative evidence and statistics
	Collate Community Health Profile	Use the scoping and screening checklists as a guide to gather data to identify relevant health intelligence and demographic, economic, environmental and social data / statistics. This includes gathering data in relation to population groups affected and determinants of health identified to be synthesised into a summary for the final report.
	Stakeholder evidence	15 stakeholders identified as part of the Scoping Process were interviewed to identify key information, knowledge and evidence.
**3b. Appraisal of Evidence**	The evidence was assessed and characterised to identify the positive and negative impacts and form a picture of the scale, scope and duration of these. This informed recommendations and conclusion.	
**4. Reporting and Recommendations**	The final HIA report was drafted and finalised by Steering Group and published.	
**5. Review, reflection and Monitoring**	A review and evaluation of the process of carrying out the HIA is currently being undertaken.	

### Screening and scoping

A Working Group was formed the first week of the lockdown in Wales, with the aim of identifying the potential impacts of the SAH policy. It did this via the social determinants framework [4] and assessed the effect it would have across vulnerable groups in Wales for example older people, children and young people, those who have caring responsibilities and lone parents. A three-hour virtual interactive session was held utilising two validated screening checklists promoted as part of standard procedure in the Welsh HIA guidance [25]. One checklist was for population groups, for example, older people, sex / gender groups, those who have long term health conditions; and one for the determinants of health, for example, behavioural (diet/nutrition, levels of physical activity), air quality, noise, access to health care services and public transportation, food and fuel poverty [25]. Links to these are contained in Additional Table 1. The discussion was transcribed and provided direction for a literature review and the health intelligence to be collected and explored for example, population demographics for older people, statistics around numbers of key workers in Wales, and the key stakeholder groups who needed to be interviewed such as the Older Peoples Commissioner and the Children’s Commissioner in Wales [70].

A scoping checklist (link in Additional Table 1) was constructed at the same time for the HIA to define the type of HIA to be undertaken (for example the use of mixed method evidence collection) and stakeholder engagement measures. The timeframes for the HIA were extremely narrow as although the HIA would identify the impacts of the policy decision, it could also provide useful evidence and information to inform policy responses to anticipated future pandemic waves.

### Appraisal

A wide range of evidence was gathered. A community health and demographic profile was constructed using data from sources such as the Wales Health Survey [71] and the Welsh Index of Multiple Deprivation [72]. This included demographic statistics such as levels of community deprivation, numbers of older people, those with caring responsibilities, children and young people and related social, physical and mental well-being levels in Wales. It also captured health intelligence around levels of mortality and morbidity for conditions such as obesity and respiratory illnesses (which are risk factors for COVID-19) and data around digital use in Wales and intersected these with the population groups. This was captured via health intelligence and other databases in Wales for example, the Welsh Index of Multiple Deprivation (links and examples are contained in Additional Table 1).

A literature review of both academic and grey literature was carried out using the search terms; social and physical distancing; quarantine, social or wider determinants of health, inequalities, outbreaks and pandemics and Wales. Academic databases searched included HMIC, Medline and PsycInfo. Criteria for the review included papers that had been published in the last 15 years in the English language. In total, 49 papers were included in the review. Most of the papers identified focussed on previous outbreaks such as Severe Acute Respiratory Syndrome (SARS) or Middle East Respiratory Syndrome (MERS), on the impact of quarantine and isolation in response to an outbreak and psychosocial impact.

In total, 15 key stakeholder representatives were approached to participate in semi-structured interviews. Nine stakeholders were interviewed, four provided written responses and two declined with no reason being provided. Stakeholders were drawn from a range of sectors, disciplines and population groups including from environmental and public health, housing, criminal justice, third sector organisations, employer and employee groups and older and young people representatives. Semi-structured interview schedules included questions on whether organisations had a programme of work in relation to COVID-19 and the SAH policy; what were the key issues their organisation and their service users were facing; had any positives been noted; and which population groups and health outcomes were impacted. Interviewees were also asked to provide any relevant published evidence and data which could inform the HIA. The responses were transcribed, assigned a number and returned to the interviewee to be validated and / or amended. The responses were then locked in a PDF and stored on a password protected drive. Further detail on questions included can be found in the published HIA report [73].

The quantitative and qualitative data and evidence was triangulated and synthesised. Peer reviewed academic publications and health intelligence were weighted with more significance, followed by grey literature and stakeholder feedback obtained via the interviews. The evidence was then analysed and viewed through the lens of the two standard validated checklists used for the Screening session [25] and characterised for impact. The impacts were classified as follows – major, moderate, minimal; short, medium and long term; positive or negative; and confirmed, probable and possible. The definitions of these are provided in Table 3.

**Table 3:** Characterisation of impact – staying at home and social distancing HIA

Impact type:	
Positive	Impacts that are considered to improve health status or provide an opportunity to do so.
Negative	Impacts that are considered to diminish health status.
Significance/intensity:	
Minimal	Of a minimum amount, quantity or degree, negligible.
Moderate	Average in intensity, quality or degree.
Major	Significant in intensity, quality or extent. Significant or important enough to be worthy of attention, noteworthy.
Duration/timeframe	
Short term	Impact seen in 0–1 year.
Medium term	Impact seen in 1–5 years.
Long term	Impact seen in over 5 years.
Likelihood:	
Possible	May or may not happen. Plausible, but with limited evidence to support.
Probable	More likely to happen than not. Direct evidence but from limited sources.
Confirmed	Strong direct evidence e.g. from a wide range of sources that an impact has already happened or will happen.

A thematic analysis was completed by the HIA lead which was discussed with, validated and agreed by the Working Group. The appraisal identified the key determinants of health that the policy would impact and the populations who would be susceptible to the impact of the SAH policy. Once completed, the HIA draft report was quality assured through several rounds of external and internal feedback from multidisciplinary public health specialists and those who were interviewed. Amendments were made to the report based on the comments received.

### Reporting

The HIA findings were presented through a report that contained the findings, recommendations, methodology, evidence, data and checklists used as part of the assessment [73]. The impacts were collated into report sections which mirrored key Welsh Government policy areas so that the impacts could be viewed at speed by the relevant decision and policy makers in a variety of sectors. The policy areas included: health and social care; business, the economy and innovation; education, children and young people and equality and justice. The report was designed interactively with clickable links throughout the document so that all sections of the report could be accessed and read quickly and easily by policy and decision makers from all sectors. It was published on the Public Health Wales and WHIASU websites and disseminated widely via a number of relevant networks. Links to this are contained in Additional Table 1.

### Review, reflection and monitoring

A ‘Review and Reflection’ meeting was held 8 weeks post publication to allow for the team to reflect on their experiences and for any immediate impact of the report and HIA to become apparent. The key questions discussed were: what worked, what could have been improved, what had the impact been to date and what could the team do differently next time. Monitoring of the impacts of the HIA report started (short to long term) in terms of influencing cross sector policy and planning, whether the recommendations were implemented, whether learning from the HIA was used to inform further HIA work and whether evidence continued to emerge to support or contradict the findings of the HIA. For example, there was evidence published at the start of the lockdown which indicated that levels of domestic abuse were increasing under the SAH policy and this was subsequently borne out by domestic abuse organisations and police forces in Wales and the UK who reported increased calls to them [74].

## Results

The aim of this paper is to outline how HIA has been used to better understand the implications of a policy decision, rather than to comprehensively describe the results of the HIA. To do this, it is necessary to outline the main impacts identified and the groups who were affected by the SAH policy.

The HIA identified a breadth and depth of impacts of the policy across the plethora of determinants of health and population groups – some of which had previously been unknown or hidden. A key positive impact identified included the compliance with the legislation to remain at home and socially distance to reduce transmission and to prevent the NHS in Wales from exceeding capacity. Other major positive impacts included a rise in the numbers of volunteers with 27% of the Welsh population stating that they were actively volunteering during lockdown [75]. In addition, evidence indicated an increase in social mobilisation and cohesion [75] and a positive effect on the environment as air quality improved in some areas of Wales [76–78]. Home working was identified as a major enabler for those who could work from home to continue to do so. In addition, it ensured that services continued to be provided to a wide range of sectors including health and social care sector and other public services. A survey carried out at the time highlighted that 44% of the Welsh working population were working from home [75].

The HIA also highlighted major negative unintended impacts of the policy on the lives and livelihoods of all members of society. These included the detrimental effect on the Welsh economy due to the closure of some sectors, for example, non-essential retail, leisure and hospitality and those employed in these sectors [79–81]. The education of children and young people was also disrupted as they were required to stay at home and be home schooled. There was a major negative impact identified for individual and societal mental health and well-being for example, individuals who worked in public and patient services and experienced fear of infection and increased pressure, stress or anxiety [75]; those who lived and worked on their own and who had their face-to-face connectedness to family, communities and social networks reduced [82]. The uncertainty during the pandemic also affected people with many reporting increasing anxiety, loneliness and social isolation [75].

Furthermore, the HIA identified a range of opportunities that could be maximised and built on in the future. The movement towards working at home was noted as opening up future opportunities for sections of the workforce to continue to work from home and this could enable them to have a better work / life balance and manage caring responsibilities [83]. It provided an opportunity for employers and employees to utilise new ways of working, increase productivity and reduce commuting time which could be better for the environment.

HIA also provides evidence about the impact of a policy decision on inequalities across the population, with some groups being disproportionately negatively affected by the policy and some benefitting. In this HIA, major negative impacts were identified for older people, babies, children and young people (0–25 years age range), key workers such as health and social care workers, those on low incomes and those with caring responsibilities including childcare. Older people and women were affected in a multi-faceted way. In total, 47% of employed women in Wales work in education, childcare, health, social care and retail sectors [84]. Many women particularly those with children also work in low paid, part time employment within the sectors closed down and largely provided caring responsibilities including home schooling.

The HIA explicitly demonstrated the difference between those who were at increased risk of contracting and dying from COVID-19 and the rest of the population who were, and could be, substantially affected via the wider determinants of health and increasing the inequality gap. For example, babies, children and young people are acknowledged to not be at major risk of mortality and morbidity but the HIA identified them as being severely affected by the SAH policy as education, social interaction and sources of employment for those working in shut down sectors was disrupted [84, 85]. A comparison is depicted in Table 4.

**Table 4:** Population Groups most affected by COVID-19 and those most affected by the lockdown and SAH Policy

Population Groups	Those at risk of direct harm of mortality and morbidity from COVID-19	Most at risk from the SAH policy to address the COVID-19 pandemic and reduce transmission
The whole population		✓
Older people	✓	✓
Men	✓	✓
Black and Minority Ethnic groups (including some Refugee and Asylum Seeker groups)	✓	✓
Those who live in areas of deprivation	✓	✓
Those who live in care homes	✓	✓
Key workers	✓ (Health care workers)	✓
Women		✓
Babies, children and young people		✓
Those who have existing mental health conditions		✓
Carers and those with caring responsibilities		✓
Those with physical and learning disabilities	✓ (Learning disabilities)	✓
Refugees and Asylum Seekers		✓

## Discussion

### Summary of results

The SAH HIA is to date the only HIA carried out on a national COVID-19 related measure such as lockdown. There are transferrable learnings for international, national, regional and local authorities and PHIs who are considering using or promoting HiAP through HIA as a process to understand the wider impact, and harms and benefits of policy decisions [86]. This paper helps to enable learning, mitigation of harm and development of interventions to aid societal recovery and increasing equity from an event such as the COVID-19 pandemic [58]. It aims to contribute to closing the knowledge gap in relation to the role of PHIs in advocating or using HIA as a tool and platform for providing evidence to inform policy and decision makers. This is little addressed in the peer reviewed literature beyond their role in capacity building [40] where the focus on the remit of PHIs is in respect to health information, data and evidence gathering and dissemination, infection and outbreak control, laboratory management and health promotion and improvement programmes such as smoking cessation and obesity interventions [87].

### The use of HIA in the decision-making process

HIA is not the only process which can be utilised to appraise the impact of policies and include health input or determinants to some extent [30, 32, 33] nor is it unusual in analysing the effect of a policy. However, it is the only IA which is explicit in its focus on health and well-being carried out via a systematic, participatory process which characterises the scale and scope of any identified impact. Being able to assess, understand and communicate the wider population impacts, beyond the direct physical health harms, of policies is important as it enables a wide range of organisations and decision makers to better understand the differential effect of policies on societal groups and communities. This is relevant to both health and non-health stakeholders due to the acknowledged importance that wider sectors and social determinants have on population health and equity [5, 6]. For example, children and young people have had their education disrupted which could affect their future economic life choices and chances and social development. In addition, their mental well-being may be affected by being isolated or disconnected from their face-to-face social networks [88, 89]. Evidence presented by this HIA could enable policy and decision makers to target action in order to address or mitigate for these. For example during the second short ‘Firebreak’ lockdown in Wales in October 2020, schools and colleges remained opened and two households were allowed to join to form an extended household to help to reduce social isolation for vulnerable people [90]. The findings can also be used to address any inequalities created or exacerbated in the short to long term [15].

The HIA allowed cross-sector and cross-determinant impact to be harnessed and collated in one report to enable a better understanding of the wide-ranging implications. It identified future actions, for example, monitoring the impact of the policy on excess morbidity and mortality, and ongoing mental health and well-being support for the population that could be implemented to protect and mitigate harm to health and well-being which are important for future health outcomes [58]. The HIA also highlighted the multifaceted, complex nature of the impact for inequalities on groups such as older people or BAME groups [91]. The HIA has been utilised to inform actions, research and plans in Wales for example, Public Health Wales is undertaking a Mental Well-being Impact Assessment (MWIA) specifically appraising the impact of the COVID-19 pandemic on children and young people in Wales (forthcoming); Public Health Wales’ Operational plan 2021–22 [92]; Welsh Government’s Remote Working programme; and a paper in relation to the lockdown, pandemic and the food environment in the United Kingdom [93]. The HIA was presented to Welsh Government ministers and heads of NHS Health Boards in Wales and shared with other national and international ministries of health, PHIs and agencies and acknowledged to be of value [94]. The HIA has also been submitted to calls for evidence in the UK about the effect of the pandemic [95–97] and as a platform for other nations to build on.

### The role of HIA by public health institutes

Whilst PHIs have a key role in protecting, improving and promoting health, there is little consideration and evidence about their role in HIA beyond advocacy and capacity building for example, carrying out HIAs [25, 40, 98, 99]. In the case of the SAH policy, it demonstrated that HIA could be used and adapted as part of emergency planning and supporting measures as a way of establishing the health, inequality and well-being impacts and / or anticipating the impact of future identical or similar situations along with other IA processes as noted by Tajima et al. [48]. PHIs also hold health intelligence required for such a HIA, for example the Public Health Observatory at Public Health Wales. In addition, PHIs can facilitate dedicated resources such as WHIASU in order to carry out or advocate for HIAs as part of a HiAP approach. This is seen as a key element of capacity building and raising awareness and ‘buy in’ for HIAP and HIA in public health and wider systems [59]. The implementation of the HIA was supported by the enabling legislation of Welsh Government but also by the proactive leadership of WHIASU which is part of Public Health Wales and can be a key enabler of HIAP [59]. Considering the impact of COVID-19 and pandemic measures across the whole of society is a key focus and third pillar of Welsh Government’s recovery framework [100] as well as Public Health Wales’ Operational Plan and associated campaigns [101]. Learning by doing is acknowledged as an important aspect of HIA capacity building [99]. This HIA provided an opportunity for capacity building and enabled public health officers to be part of a collaborative HIA of a rare event in modern times and build capacity, skills and knowledge in a safe space [14, 99].

HIA is already acknowledged in the literature as a useful tool to apply in order to anticipate impacts in a prospective and balanced way which it does by identifying both positive and negative impact [23]. This HIA demonstrated that there is also value in carrying out HIAs to explicitly articulate impacts as they occur. The HIA was carried out alongside the health protection response and collected evidence in ‘real time’ as it emerged and has added value to future policy decisions by raising awareness explicitly of the health and population impacts of those decisions as they occur which has been noted by others [15, 16, 50]. HIA can be utilised in times of unprecedented situations such as health (or other kinds of) emergencies. It can also be used when decisions need to be made at speed to protect the public and their health or safety for example, a lockdown and / or where there are no previous examples or when there is little evidence to assist the decision-making process for example, policy and political decisions such as Brexit in the UK [99].

### Constraints and limitations

The SAH HIA did face some constraints and limitations. It was carried out in ‘real time’ against a dynamic and evolving situation which meant that the impacts and findings needed to be reviewed and amended if required until the publication date. The team aimed to produce a high quality report in a timely manner in order to provide information and evidence for decision makers. With unique events such as Brexit or unprecedented responses to events such as the COVID-19 pandemic, there may be evidence in existence or available but not for all determinants or groups. There is now a raft of evidence in relation to the health and wider impacts of the COVID-19 pandemic in Wales [62–66](refs) which researchers and impact assessment practitioners and policy makers can draw on. The lack of evidence of previous extensive and comprehensive measures across the population highlighted some areas which needed to be monitored and explored further, for example the impact on refugees and asylum seekers in Wales. This had been identified as part of the stakeholder interviews and deemed important. A specific review and reflection session enabled the team to capture learning in order to support the evolution of HIA practice in Wales. This paper shares some of this learning but it is also useful for HIA and IA practitioners and processes to learn from each other around how they include health and well-being [33, 34].

### Future research

Finally, the HIA flagged up gaps in the evidence base that require further exploration of both the short and longer-term impact of the lockdown policy implemented in response to the pandemic. These include the multifaceted impact on particular population groups such as children and young people, and women; the need for more research on the impact of such a situation on health behaviours including diet and physical activity, violence against women, domestic abuse and sexual violence (VAWDASV) and the mental health and well-being impact across a number of population groups. This has led to the commissioning of several work streams on some of these topics in Public Health Wales including a systematic literature review and the MWIA. These were both recommendations in the HIA report. The HIA also highlighted the need to carry out further HIA work to explore particular aspects and interventions that were component parts of the Coronavirus legislation such as the accelerated move to more employees’ home working [72] and to explore further how it has been utilised by policy makers to understand and react to the impacts and inequalities identified.

## Conclusions

HIA has a key role in policymaking and can support PHIs, regional and national governments in their roles to promote and protect health and well-being, and reduce the inequality gap. HIA can do this by enabling both health and ‘non-health’ stakeholders to better understand the wider health impact of a policy which has been implemented at speed. It can explicitly identify a wide range of positive intended and negative unintended health implications for populations as well as societal, economic and environmental well-being.

The SAH HIA demonstrates how the process is a beneficial tool to inform and understand a policy decision and the unknown short and long-term challenges which emergency and unpredicted major events such as the COVID-19 pandemic present. These can be captured using a ‘real time’ HIA approach that identifies the impacts as they emerge so that that future policies and plans can be adjusted to mitigate for negative health impacts and maximise positive impacts. The impacts identified can add to the evidence base in relation to the wider impact of COVID-19 and the HIA can be utilised by policy and decision-makers and the HIA community in the future to inform, promote or carry out similar HIAs.

The HIA involves key cross sectoral and multi-disciplinary stakeholders and evidence and can enable an evidence based HIAP approach. The HIA of the SAH policy in Wales has transferrable learnings in relation to the use of HIA in promoting a better understanding of the immediate and the long-term ramifications of policy decisions but also raises awareness of how PHIs can use HIA to communicate any harm to, and opportunities for, population health and well-being in order to advocate for healthier and fairer societies.

## Supplementary Information

**Additional file 1 Table S1.** The Methodological Process for the SAH HIA.

## Data Availability

The datasets used and/or analysed during the current study are available from the corresponding author on reasonable request.
