# Supplementary material for: Using health impact assessment (HIA) to understand the wider health and well-being implications of policy decisions: the COVID-19 ‘staying at home and social distancing policy’ in Wales
Source: BMC Public Health. 2021 Jul 27;21:1456. doi: 10.1186/s12889-021-11480-7 (PMC8313659; doi:10.1186/s12889-021-11480-7)
Supplement: Supplementary file 1 — Additional file 1 Table S1. The Methodological Process for the SAH HIA. [file 12889_2021_11480_MOESM1_ESM.docx]

**Additional Table 1: The Methodological Process for the SAH HIA**

| **HIA Step** | **Actions** | | **How Step was undertaken in the SAH HIA** | **Useful links for further information** |
| --- | --- | --- | --- | --- |
| This HIA was undertaken between April 2^nd^ 2020 and May 11^th^ 2020. | | | | |
| 1. **Screening** | Identify the preliminary health and well-being impact and those affected in the population. | | - A Working Group was established internal to Public Health Wales. - Wide ranging populations and determinants affected were identified. - 2 checklists were used as a guide. | <https://whiasu.publichealthnetwork.cymru/files/6415/9430/8755/HIA_-_Rapid_Review_of_SAH_Policy_Supporting_Info.pdf>  See Section 5, page 52  [WHIASU_Population_Groups_Checklist.pdf (publichealthnetwork.cymru)](https://whiasu.publichealthnetwork.cymru/files/5815/8408/9421/WHIASU_Population_Groups_Checklist.pdf) |
| 1. **Scoping** | Use a Scoping checklist to identify the:   - geographical boundaries of the HIA, methods of evidence collection key stakeholders to be involved and resources required - timeframes / deadlines - characterise and define the impact terminology for example, major, long term, positive | | - Wales is the boundary - Literature review of journal papers and grey literature to be carried out - Health intelligence and other statistics and data to be collated - Interviews with key stakeholders to be carried out - HIA Team is the resource - Timeframe was as soon as possible to inform policy and decision makers | Internal use only.  Checklist template example available at: <https://whiasu.publichealthnetwork.cymru/files/6414/9993/9603/Scoping_info.pdf> |
| **3a. Appraisal –**  **Evidence Gathering** | Literature Review | Carry out literature review and synthesise into summary to identify relevant qualitative and quantitative evidence and statistics | Search terms used included: social and physical distancing; quarantine, social or wider determinants of health, inequalities, outbreaks and pandemics and Wales. Criteria for the review included papers that had been published in the last 15 years in the English language. Most of the papers identified focussed on previous outbreaks such as Severe Acute Respiratory Syndrome (SARS) or Middle East Respiratory Syndrome (MERS), on the impact of quarantine and isolation in response to an outbreak and psychosocial impact. | <https://whiasu.publichealthnetwork.cymru/files/6415/9430/8755/HIA_-_Rapid_Review_of_SAH_Policy_Supporting_Info.pdf>  See Section 3, page 55 |
|  | Collate Community Health Profile | Use the scoping and screening checklists as a guide to gather data to identify relevant health intelligence and demographic, economic, environmental and social data / statistics. This includes gathering data in relation to population groups affected and determinants of health identified to be synthesised into a summary for the final report. | Examples of websites accessed:   - Welsh Government - Welsh Health Survey - Welsh Health Observatory - Welsh Index of Multiple Deprivation - Office of National Statistics - Statistics Wales   All data collected was compiled into a Community Health Profile which fed into the final HIA report. | <https://whiasu.publichealthnetwork.cymru/files/6415/9430/8755/HIA_-_Rapid_Review_of_SAH_Policy_Supporting_Info.pdf>  See Section 2, page 8 |
|  | Stakeholder evidence | Identify key information, knowledge and evidence of external stakeholders identified as part of the Scoping Process by:   - Inviting identified stakeholders to be interviewed - Developing topic guide and participant information - Undertaking interviews and transcribing for analysis - Developing a summary of results to input into the final report. | 13 stakeholder interviews were undertaken, which included for example:   - Natural Resources Wales - Public Health Wales Healthy Working Wales Team - Trade Union Congress Wales - Public Health Wales Healthy Schools Programme Lead - Public Health Wales Environmental Health Protection Consultant - Children’s Commissioner for Wales - Office of the Future Generations of Wales Commissioner - Welsh Local Government Association - Welsh Government | <https://whiasu.publichealthnetwork.cymru/files/6415/9430/8755/HIA_-_Rapid_Review_of_SAH_Policy_Supporting_Info.pdf>  See Section 4, page 49 and the acknowledgements |
| **3b. Appraisal of Evidence** | - Assess and characterise the positive and negative impacts and form a picture of the scale, scope and duration of these. - Form recommendations and conclusion | | Impact on determinants of health identified included, for example:   - Volunteering and social mobilisation <https://phw.nhs.wales/topics/latest-information-on-novel-coronavirus-covid-19/how-are-you-doing/how-are-we-doing-in-wales-reports/week-25-report-how-are-we-doing-in-wales/> - Domestic violence <https://www.ons.gov.uk/peoplepopulationandcommunity/crimeandjustice/articles/domesticabuseduringthecoronaviruscovid19pandemicenglandandwales/november2020> - Home working - Mental well-being <https://www.mentalhealth.org.uk/our-work/research/coronavirus-mental-health-pandemic> - Environment <https://airquality.gov.wales/sites/default/files/documents/2020-08/Analysis_of_Welsh_Air_Quality_Data_Impacts_of_Covid-19_Final_Issue2.pdf> . - Economy <https://seneddresearch.blog/2020/10/13/coronavirus-youth-unemployment/>   Population Groups identified as being affected included for example:   - Children and young people <https://www.nspcc.org.uk/about-us/news-opinion/2020/childline-lifeline-coronavirus/> . - Those on low incomes <https://www.ifs.org.uk/publications/14791> - Black, Asian and Minority Groups <https://assets.publishing.service.gov.uk/government/uploads/system/uploads/attachment_data/file/908434/Disparities_in_the_risk_and_outcomes_of_COVID_August_2020_update.pdf> - Those with mental health conditions <https://www.euro.who.int/en/health-topics/health-emergencies/coronavirus-covid-19/news/news/2020/3/mental-health-and-psychological-resilience-during-the-covid-19-pandemic> - Characterisation of Impact table drafted. | <https://whiasu.publichealthnetwork.cymru/files/6415/9430/8755/HIA_-_Rapid_Review_of_SAH_Policy_Supporting_Info.pdf>  See Section 3, page 10 and also:  <https://whiasu.publichealthnetwork.cymru/files/3915/9280/5148/HIA_-_Rapid_Review_of_SAH_Policy_Exec_Summary.pdf>  See Table 1, page 11 |
| **4. Reporting and Recommendations** | - Compile report and recommendations. - Carry out quality assurance with key stakeholders - Review and Sign off in NPHI - Publication of HIA | | - Report published - Press release published - Dissemination via networks and stakeholders involved | <https://whiasu.publichealthnetwork.cymru/files/3915/9280/5148/HIA_-_Rapid_Review_of_SAH_Policy_Exec_Summary.pdf>  <https://whiasu.publichealthnetwork.cymru/files/4515/9618/5918/HIA_-_Rapid_Review_of_SAH_Policy_Main_Report.pdf>  Future actions – See Section 5, page 84 |
| **5. Review, reflection and Monitoring** | - Review and evaluate the process of carrying out the HIA - Develop monitoring indicators / steps | | Compile review and reflection paper.  Ongoing. | Internal use only |
